# Neighborhood Socioeconomic Deprivation and Mortality: NIH-AARP Diet and Health Study

**DOI:** 10.1371/journal.pone.0015538

**Published:** 2010-11-23

**Authors:** Jacqueline M. Major, Chyke A. Doubeni, Neal D. Freedman, Yikyung Park, Min Lian, Albert R. Hollenbeck, Arthur Schatzkin, Barry I. Graubard, Rashmi Sinha

**Affiliations:** 1 Division of Cancer Epidemiology and Genetics, National Cancer Institute, National Institutes of Health, Bethesda, Maryland, United States of America; 2 Department of Family Medicine and Community Health, University of Massachusetts Medical School, Worcester, Massachusetts, United States of America; 3 Department of Medicine, Washington University School of Medicine, St. Louis, Missouri, United States of America; 4 American Association of Retired Persons, Washington, District of Columbia, United States of America; Yale University School of Medicine, United States of America

## Abstract

**Purpose:**

Residing in deprived areas may increase risk of mortality beyond that explained by a person's own SES-related factors and lifestyle. The aim of this study was to examine the relation between neighborhood socioeconomic deprivation and all-cause, cancer- and cardiovascular disease (CVD)-specific mortality for men and women after accounting for education and other important person-level risk factors.

**Methods:**

In the longitudinal NIH-AARP Study, we analyzed data from healthy participants, ages 50–71 years at study baseline (1995–1996). Deaths (n = 33831) were identified through December 2005. Information on census tracts was obtained from the 2000 US Census. Cox models estimated hazard ratios (HRs) and 95% confidence intervals (CIs) for quintiles of neighborhood deprivation.

**Results:**

Participants in the highest quintile of deprivation had elevated risks for overall mortality (HR_men_ = 1.17, 95% CI: 1.10, 1.24; HR_women_ = 1.13, 95% CI: 1.05, 1.22) and marginally increased risk for cancer deaths (HR_men_ = 1.09, 95% CI: 1.00, 1.20; HR_women_ = 1.09, 95% CI: 0.99, 1.22). CVD mortality associations appeared stronger in men (HR = 1.33, 95% CI: 1.19, 1.49) than women (HR = 1.18, 95% CI: 1.01, 1.38). There was no evidence of an effect modification by education.

**Conclusion:**

Higher neighborhood deprivation was associated with modest increases in all-cause, cancer- and CVD-mortality after accounting for many established risk factors.

## Introduction

During the past three decades a number of studies have examined the impact of socioeconomic status (SES) and SES-related factors on mortality. Consistent associations with increased risk of total mortality as well as cause-specific mortality have been reported for person-level SES measures [1], [2], [3], [4], [5], [6], [7]. Yet the mechanisms linking SES to mortality are not clear. Surprisingly, only a limited number of large geographically diverse longitudinal studies have examined these associations on the U.S. population. Most of these have focused on all-cause mortality with men and women combined and have examined SES at the person- or household-level (e.g., education, race, household income, etc.). As few studies possess both person-level and area-level data, the interaction between these measures, though potentially of significant importance, is poorly understood [8], [9], [10].

The large longitudinal NIH-AARP Study provides a rich resource to personal characteristics and health risk factors and is linked to census tract data which details the composition and socioeconomic context of each participant's local environment. The purpose of the present study was to examine the relation between neighborhood deprivation and all-cause, CVD- and cancer-related mortality for men and women after adjusting for a broad array of important risk factors.

## Materials and Methods

### Study population

From 1995 through 1996, men and women between the ages of 50–71 years residing in one of six U.S. states (California, Florida, Louisiana, New Jersey, North Carolina, and Pennsylvania) or two metropolitan areas (Atlanta, Georgia, and Detroit, Michigan) were recruited to participate in the NIH-AARP Diet and Health Study, a large prospective cohort study examining the relation between diet and health [11]. The NIH-AARP Diet and Health Study was approved by the Special Studies Institutional Review Board of the U.S. National Cancer Institute, and written informed consent was obtained from all participants by virtue of completing the baseline questionnaire.

Our baseline cohort included 566,402 persons. We excluded individuals whose questionnaire was completed by someone else on their behalf (n = 15,760), subjects who reported having end-stage renal disease, previous cancer, heart disease, stroke or emphysema (n = 136,785), and subjects reporting extreme daily total energy intake defined as more than two inter-quartile ranges above the 75th percentile or below the 25th percentile (n = 3583). In addition, 495 individuals were excluded due to missing census information. Individuals were also excluded if their date of death or dropout preceded the date of study entry (n = 3 and n = 1, respectively). After exclusions, our analytic cohort consisted of 233,205 men and 176,570 women.

### Study measures

#### Vital status and cause-specific mortality

Vital status was ascertained through linkage to the Social Security Administration Death Master File and National Death Index from recruitment (1995–1996) to December 31, 2005. In addition to all-cause mortality (n = 33,831), we investigated the two leading causes of death: cardiovascular disease (n = 8,952) and cancer (n = 14,091). Details on case ascertainment have been described elsewhere [12].

#### Neighborhood socioeconomic deprivation index

Information on neighborhood characteristics was obtained from the 2000 US Census. In the present study, census tracts (n = 18603) were used as proxies for neighborhoods. NIH-AARP participants were linked to their affiliated census tract by their home address.

Following the approach outlined in detail by Messer and colleagues [13], we constructed a deprivation index using principal components analysis (PCA). In brief, nineteen census tract-variables representing seven contextual factors of neighborhood environment (housing, residential stability, poverty, employment, occupation, racial composition, and education) were considered for inclusion in the index. The resulting first principal component was examined to identify higher-loading variables (i.e., variables that loaded above 0.25) in each state and then assessed for consistency across states pooled together. Based on the results from PCA, ten variables were retained for the index: % total with less than high school, % non-Hispanic blacks, % total unemployed, % females in management, % males in management, % households with income (1999) below poverty, % female head of household, % households with public assistance income, % households with income <30 k, % households with no vehicle. The PCA was repeated and final loadings for the ten variables were used to calculate a deprivation summary score for each census tract [13].

#### Demographics and behavioral risk factors

Information on individual-level demographic and health-related behaviors was ascertained through a mailed survey sent to members of AARP at baseline in 1995. This survey included a 124-item food frequency questionnaire (FFQ) that collected information on a participant's usual dietary intake within the past 12-month, including portion sizes, (http://www.risk.factor.cancer.gov/DHQ) as well as information on lifestyle and medical history. Daily intake of red and white meats and fruits and vegetables were energy-adjusted using the nutrient density method [14]. Physical activity was assessed on the baseline questionnaire by asking subjects how often they participated in physical activities at work or home, including exercise, sports, and activities such as carrying heavy loads during a typical month in the prior 12 months. Frequency of vigorous physical activity was defined as 5 or more times per week. Self-reported body weight (lbs) and height (ft-inches) were used to derive body mass index [weight (kg)/height (m^2^)].

### Statistical analysis

Descriptive statistics were calculated for baseline characteristics of participants by gender. General linear models were used to examine age-adjusted characteristics across quintiles of deprivation. Quintiles for person-level variables were based on the full study cohort; quintiles for deprivation index were based on the distribution across census tracts. Separately in men and women, multivariable Cox regression models with a robust variance estimator that accounts for the correlation of individuals residing within the same census track was used to assess associations between neighborhood deprivation quintiles and all-cause as well as cause-specific mortality [15], [16]. We examined the within and between components of variability using Cox models with gamma frailties [17], [18], which gave almost identical estimates of relative hazards due to the between-neighborhood variability being small, therefore findings from the former models are reported in the present study. Time since entry into the study was used as the underlying time metric. Participants were followed from the date the baseline questionnaire was returned to the date of death or the end of study follow-up (December 31, 2005), whichever came first. Potential interactions were evaluated using cross-product terms along with the main effects in the model. The hazard ratio (HR) and 95% confidence interval (CI) were calculated for each variable in the Cox models. Fully adjusted models included age (continuous), education, race/ethnicity, marital status, family history of cancer (for cancer mortality only), body mass index (<18.5, 18.5 to <25, 25 to <30, ≥30 kg/m^2^), smoking (never, former ≤20 cigs/d, former >20 cigs/d, current ≤20 cigs/d, current >20 cigs/d, missing), vigorous physical activity (≥5 or more times/week), self-reported health status (excellent/great, good, fair/poor), vitamin or mineral use (≥once a month), menopausal hormone use (for women only), total energy intake (continuous), alcohol intake (none, 0 to <5, 5 to <15, 15 to <30, ≥30 g/day), and intakes of red meat, white meat, fruits, and vegetables (g/1000 kcal categorized into quintiles) by progressively introducing the variables in the model. The test for trend across deprivation quintiles was performed by determining the quintile-specific median values and entering that variable as a continuous term in the regression model. Statistical significance was based on two-sided *P* values of <0.05. Data were analyzed separately for men and women using SAS® (version 9.2, SAS Institute Inc., Cary, NC), and Survival package in R software (version 2.10) was used for the Cox regression with gamma frailties modeling.

## Results

During the median follow-up period of 9.5 years, approximately 9% (n = 21843) of men and 7% (n = 11988) of women died. The leading causes of mortality were cancer and cardiovascular-related disease, comprising almost 70% of total deaths.

Subject characteristics were examined across quintiles of deprivation (Tables 1 and 2). In our study population, 6% men and 9% women resided in the most deprived areas (deprivation quintile 5). In general, those in the highest quintile of deprivation were more likely to be of African-American descent or of another non-white race, less educated, unmarried, current smokers, have higher daily intakes of total energy and carbohydrates, consume less alcohol per day, and less likely to report being in good health or use vitamin supplements. In addition, women in the most deprived areas were less likely to be physically active or to be using hormones at baseline.

**Table 1:** Age-adjusted characteristics across quintiles of the deprivation index (Men, N = 233,205).

		Deprivation Index Quintiles		
Sex	Parameter	Least deprivedQ1	Q3	Most deprivedQ5
Men	Participants, n	77132	49366	13686
	Total deaths, %	7.4	10.4	12.3
	CVD deaths, %	1.9	2.9	3.8
	Cancer deaths, %	3.2	4.2	4.5
	Mean deprivation	−1.2	−0.2	1.3
	Age, years	61.4	62.0	61.6
	Race, %			
	Non-Hispanic white	94.7	93.2	70.6
	Non-Hispanic black	0.9	2.1	20.8
	Other	4.4	4.7	8.6
	Currently married, %	88.5	84.6	72.3
	Family history of cancer, %	48.3	46.8	43.4
	BMI, kg/m^2^	26.8	27.5	27.7
	Smoking history, %			
	Never	37.0	29.7	30.3
	Former	53.9	56.4	51.7
	Current or quit <1 yr	9.0	14.0	18.0
	Education, college grad+, %	64.1	35.1	29.6
	Physical activity 5+ times/wk, %	21.5	20.9	19.1
	Health status, %			
	Excellent/great	68.6	57.7	49.4
	Good	27.2	34.9	38.6
	Fair/poor	4.2	7.4	12.0
	Dietary intake			
	Energy, kcal/d	1961	2069	2131
	Fruit, srv/1000 kcal	1.6	1.5	1.6
	Vegetables, srv/1000 kcal	2.1	2.0	1.9
	Alcohol, g/d	17.6	16.8	15.7
	Saturated fat, g/1000 kcal	10.4	10.9	10.9
	Carbohydrates	246	258	271
	Cholesterol, mg	216	237	250
	Vitamin or mineral use ≥1/mo	61.0	56.6	52.1
	Meat intake			
	Red meat, g/1000 kcal	36.5	40.1	38.9
	White meat, g/1000 kcal	34.3	29.5	29.9

Data are given as mean value or percentage of participants. Generalized linear models were used to estimate age-adjusted mean values for continuous variables and logistic regression for proportions within each deprivation quintile. There were approximately 3720 census tracts within each deprivation quintile.

**Table 2:** Age-adjusted characteristics across quintiles of the deprivation index (Women, N = 176,570).

		Deprivation Index Quintiles		
Sex	Parameter	Least deprivedQ1	Q3	Most deprivedQ5
Women	Participants, n	47894	39549	15749
	Total deaths, %	5.5	7.1	8.7
	CVD deaths, %	1.2	1.7	2.2
	Cancer deaths, %	2.7	3.0	3.5
	Mean deprivation	−1.2	−0.2	1.4
	Age, years	61.2	61.9	61.7
	Race, %			
	Non-Hispanic white	94.2	92.4	58.5
	Non-Hispanic black	1.4	3.0	33.0
	Other	4.4	4.6	8.4
	Currently married, %	49.0	45.4	34.6
	Family history of cancer, %	51.8	51.6	47.6
	BMI, kg/m^2^	25.7	27.0	28.4
	Smoking history, %			
	Never	46.0	47.4	48.6
	Former	40.8	35.7	31.9
	Current or quit <1 yr	13.2	17.0	19.6
	Education, college grad+, %	44.1	24.2	22.8
	Physical activity 5+ times/wk, %	18.7	15.9	13.8
	Health status, %			
	Excellent/great	65.7	54.3	43.8
	Good	28.4	36.0	40.2
	Fair/poor	5.9	9.7	16.0
	Current HRT use, %	67.0	61.1	52.5
	Dietary intake			
	Energy, kcal/d	1514	1572	1690
	Fruit, srv/1000 kcal	2.0	1.9	2.1
	Vegetables, srv/1000 kcal	2.6	2.5	2.3
	Alcohol, g/d	7.3	5.4	4.0
	Saturated fat, g/1000 kcal	10.0	10.5	10.4
	Carbohydrates	205	212	232
	Cholesterol	157	169	184
	Vitamin or mineral use ≥1/mo	71.4	66.6	58.0
	Meat intake			
	Red meat, g/1000 kcal	26.8	30.7	29.9
	White meat, g/1000 kcal	38.7	34.4	35.7

Data are given as mean value or percentage of participants. Generalized linear models were used to estimate age-adjusted mean values for continuous variables and logistic regression for proportions within each deprivation quintile. There were approximately 3720 census tracts within each deprivation quintile.

The age- and multivariable-adjusted HRs and corresponding 95% CIs are presented in Table 3. Strongest effects were observed for upper quintiles of deprivation. After age adjustment, the risk of dying was 1.66 (95% CI: 1.58, 1.75) times greater for men and 1.53 (95% CI: 1.43, 1.63) for women living in the most deprived areas compared to those living in the least deprived areas. There was no evidence of a threshold effect; instead the association between neighborhood deprivation and mortality was graded, with the risks of mortality lowest for those in the least deprived areas and then increasing with increasing quintiles of deprivation. This pattern in the age-adjusted models was similar for cause-specific mortality as well.

**Table 3:** Association between deprivation index and all-cause or cause-specific mortality.

	All-cause Mortality		CVD Mortality		Cancer Mortality	
	Age-adjusted	Multivariable-adjusted	Age-adjusted	Multivariable-adjusted	Age-adjusted	Multivariable-adjusted
Men						
Deprivation (ref: Q1)						
Quintile 2	1.21 (1.16, 1.25)	1.05 (1.01, 1.10)	1.30 (1.22, 1.40)	1.13 (1.05, 1.22)	1.19 (1.13, 1.26)	1.05 (0.99, 1.11)
Quintile 3	1.36 (1.31, 1.41)	1.11 (1.06, 1.15)	1.49 (1.38, 1.59)	1.18 (1.10, 1.27)	1.29 (1.22, 1.37)	1.07 (1.01, 1.14)
Quintile 4	1.53 (1.47, 1.60)	1.17 (1.12, 1.22)	1.82 (1.69, 1.96)	1.33 (1.23, 1.45)	1.39 (1.30, 1.48)	1.10 (1.03, 1.18)
Quintile 5	1.66 (1.58, 1.75)	1.17 (1.10, 1.24)	2.02 (1.84, 2.23)	1.33 (1.19, 1.49)	1.44 (1.32, 1.57)	1.09 (1.00, 1.20)
*P* trend	<0.001	<0.001	<0.001	<0.001	<0.001	0.006
Women						
Deprivation (ref: Q1)						
Quintile 2	1.15 (1.09, 1.21)	1.03 (0.98, 1.09)	1.19 (1.07, 1.33)	1.02 (0.91, 1.15)	1.08 (1.01, 1.17)	1.02 (0.94, 1.10)
Quintile 3	1.22 (1.16, 1.29)	1.04 (0.99, 1.10)	1.39 (1.25, 1.55)	1.13 (1.01, 1.28)	1.07 (1.00, 1.16)	0.97 (0.89, 1.05)
Quintile 4	1.37 (1.30, 1.45)	1.11 (1.04, 1.17)	1.67 (1.49, 1.87)	1.26 (1.12, 1.43)	1.12 (1.03, 1.22)	0.97 (0.89, 1.07)
Quintile 5	1.53 (1.43, 1.63)	1.13 (1.05, 1.22)	1.84 (1.61, 2.09)	1.18 (1.01, 1.38)	1.27 (1.15, 1.40)	1.09 (0.99, 1.22)
*P* trend	<0.001	<0.001	<0.001	<0.001	<0.001	0.558

HRs (95% CIs) are reported.

Deprivation quintile 1 = least deprived, quintile 5 =  most deprived.

Multivariable models were adjusted for age, education, race, marital status, family history of cancer (cancer mortality model), physical activity, smoking, self-reported health status, BMI, energy, cholesterol (CVD mortality model), alcohol use, fruit, vegetables and meat intakes, and vitamin use. Models for women were additionally adjusted for menopausal hormone use.

Observed associations with neighborhood deprivation were attenuated after adjustment for the person-level covariates with smoking being the strongest confounder; however, deprivation remained a statistically significant independent predictor of mortality. Associations were similar for men and women with the exception of men having a stronger effect of deprivation on CVD-related deaths (1.33 vs. 1.18). The risk of dying was 1.17 (95% CI: 1.10, 1.24) and 1.09 (95% CI: 1.00, 1.20) for men and 1.13 (95% CI: 1.05, 1.22) and 1.09 (95% CI: 0.99, 1.22) for women residing in the most deprived areas compared to those in the least deprived (reference category) for all-cause and cancer mortality, respectively.

Additional analyses (not shown) found no significant interactions between individual education and neighborhood deprivation. Sensitivity analyses did not show marked differences in associations when examining the relation between neighborhood deprivation and mortality within states or after stratification by smoking status.

## Discussion

In this prospective study of older U.S. adults, we found that men and women residing in the most deprived census tracts had an increased risk of dying (approximate 15% and 10% occurred for all-cause and cancer deaths, respectively) compared to those in the least deprived areas, with the greatest risks being for CVD mortality (men, 33%; women, 18%). This excess risk was observed even after accounting for a gamut of known risk factors, including age, education, smoking, physical activity and dietary intake. Our results emphasize the importance of neighborhood SES and related contextual factors for decreasing the risk of mortality from the two leading causes of death: cardiovascular disease and cancer.

Few previous studies have examined sex-specific differences in the pathways that produce socioeconomic mortality gradients. Our relative risks of CVD mortality for women residing in high deprivation areas are consistent with those reported by LeClure and colleagues for a similar age group when examining individual neighborhood characteristics, i.e., percent female headship [19]. Further, our finding of a sex difference between deprivation and CVD mortality are in accord with studies, which found that the socioeconomic-mortality association is weaker in women than men for person-level indicators of SES [1], [3], [20].

Our results are not in accord with those reported by Bosma and colleagues who examined individual neighborhood SES characteristics in a specific geographical area of the Netherlands for a wider age range, 15–74 years [21]. The lower risks observed for total mortality in our study may in part be explained by differences in the study populations, with NIH-AARP Study having less variability in SES and comprised of older adults (50–71 yrs). Stronger effects of deprivation on mortality have been reported for younger age groups [22]. Another important difference is that we accounted for a broader array of individual risk factors, such as smoking, physical activity, and dietary intake, which attenuated the relative risks.

The present study extends what has been done in previous studies by using a composite measure of neighborhood socioeconomic deprivation comprised of ten individual census variables, evaluating effects separately for men and women, examining all-cause as well as cancer and CVD-related deaths, accounting for an extensive list of individual risk factors, and is not restricted to a specific geographic area [21], [22], [23], [24], [25], [26], [27], [28], [29], [30], [31], [32], [33], [34]. Our results indicate an independent relation between area-level deprivation and mortality lending further support for future research and interventions to consider both person- and area-level factors of deprivation.

Potential mechanisms by which neighborhood deprivation may influence mortality include physical environment/environmental exposures and social norms. In particular, areas with higher deprivation may have issues such as limited availability of or access to healthy foods and health care, higher environmental pollution, and lack of social networks that might also impact mortality risk independently of the characteristics of the people living in those areas [35]. We were unable to examine the potential influence of physical environment in the present study. There was some evidence that social norms may have played a role in the increased risk of mortality since the risk estimates for deprivation were further attenuated after adjusting for behavioral risk factors that may reflect the social norms of more deprived areas (e.g., smoking, high BMI) [36], [37].

Among the inherent strengths of the present study is the prospective design in which individual- and area-level factors were measured prior to mortality. Extensive data collection of information on lifestyle and medical history allowed us to control for possible confounding on a broad array of characteristics and lifestyle factors. In addition, we employed an innovative analytic method, the extended Cox model with a robust variance estimator, to simultaneously examine associations of person-level and area-level risk factors with mortality, which is unique in research evaluating the effects of neighborhood deprivation on death. Further, the large size of the NIH-AARP Study allowed us to stratify the study population by gender and examine interactions while maintaining study power.

A limitation of our study is that the cohort was predominantly older, upper-to-middle class Caucasians; therefore, results may not apply to other populations. Yet even within the NIH-AARP study population that may have a limited range of neighborhood deprivation scores, we were still able to observe an effect of deprivation on mortality. It is possible that this effect might actually be larger for a population with a wider range of socioeconomic status. It is also possible that results for neighborhood deprivation reflect incomplete adjustment for person-level factors, including household income and occupation. Given that this is an older cohort with many retirees, individuals may have relocated after retirement and therefore the census track at study baseline may not reflect that of younger ages. Although information on long-term residency was not ascertained in the present study, the NIH-AARP did collect a wide range of characteristics and lifestyle factors, including dietary intake, that are typically not available in other study populations. Because we investigated multiple endpoints, it is possible that significant results may be due to chance. Future studies are needed to replicate these findings.

In conclusion, our data provide support for an independent relation between neighborhood socioeconomic deprivation and mortality in both men and women, even after accounting for a large number of known risk factors for mortality. Our findings require confirmation in other U.S. populations, including younger age groups and populations with a wider range of SES. If confirmed, further studies are needed to identify important pathways (e.g., access to and quality of health services, social networks, air quality, etc.) that may underlie these results. Identification of these pathways could have important public health and policy implications.
